# Supplementary material for: Are We Approaching Peak Meat Consumption? Analysis of Meat Consumption from 2000 to 2019 in 35 Countries and Its Relationship to Gross Domestic Product
Source: Animals (Basel). 2021 Dec 6;11(12):3466. doi: 10.3390/ani11123466 (PMC8697883; doi:10.3390/ani11123466)
Supplement: Supplementary file 1 [file animals-11-03466-s001.zip › animals-1457101-supplementary.pdf]

SUPPLEMENTARY MATERIAL

Supplementary Table S1. Total meat consumption in 2000 and 2019 and % change, presented for countries with decreasing, increasing and no temporal trend

Country	Total meat consumption		Change in total meat consumption		
	Year 2000	Year 2019	% change (2000 - 2019)	Unstandardised β coefficient (SE)	P value
	Kg/capita	Kg/capita	%	Kg/capita/year	
Decreasing linear trend					
Canada	76.5	70.2	-8.3	-0.49 (0.07)	< 0.001
Ethiopia	4.4	3.1	-29.8	-0.10 (0.02)	< 0.001
New Zealand	86.7	75.2	-13.2	-0.96 (0.22)	< 0.001
Nigeria	6.2	5.0	-19.2	-0.09 (0.01)	< 0.001
Paraguay	53.5	39.5	-26.2	-0.48 (0.22)	0.043
Switzerland	54.7	51.6	-5.6	-0.14 (0.06)	0.023
Increasing linear trend					
Argentina	75.6	88.3	16.8	1.34 (0.17)	< 0.001
Australia	88.2	89.6	1.6	0.28 (0.09)	0.008
Brazil	62.6	78.9	26.0	1.02 (0.11)	< 0.001
Chile	52.4	81.3	55.2	1.42 (0.08)	< 0.001
China (People's Republic of)	36.8	45.7	24.1	0.75 (0.07)	< 0.001
Colombia	27.1	48.9	80.6	1.28 (0.04)	< 0.001
Egypt	16.8	20.0	19.1	0.27 (0.04)	< 0.001
Indonesia	6.7	11.4	69.3	0.24 (0.02)	< 0.001
India	3.0	3.6	20.1	0.03 (0.004)	< 0.001
Iran	20.1	33.2	64.7	0.68 (0.04)	< 0.001
Israel	78.6	90.0	14.5	0.48 (0.14)	0.002
Japan	35.1	41.6	18.4	0.34 (0.05)	< 0.001
Kazakhstan	27.8	49.1	76.7	1.13 (0.13)	< 0.001
Korea	34.1	62.1	81.8	1.49 (0.07)	< 0.001
Malaysia	38.6	60.4	56.4	1.29 (0.05)	< 0.001
Mexico	40.6	54.6	34.7	0.59 (0.04)	< 0.001
Norway	45.7	56.0	22.4	0.60 (0.06)	< 0.001
OECD	63.0	70.1	11.3	0.24 (0.05)	< 0.001
Pakistan	9.5	15.0	57.3	0.31 (0.01)	< 0.001
Peru	25.3	54.7	116.3	1.73 (0.05)	< 0.001
Philippines	22.8	32.4	42.1	0.51 (0.02)	< 0.001
Russia	29.5	62.5	112.2	1.64 (0.12)	< 0.001
Saudi Arabia	44.0	43.6	-1.0	0.26 (0.12)	0.043
South Africa	31.9	51.6	61.7	1.28 (0.09)	< 0.001
Turkey	18.4	32.8	78.0	0.96 (0.06)	< 0.001
Ukraine	28.5	40.1	40.6	0.88 (0.15)	< 0.001
Viet Nam	19.3	50.5	161.1	2.11 (0.13)	< 0.001
World	29.5	34.0	15.3	0.30 (0.02)	< 0.001

No trend detected[a]					
Thailand	21.2	19.0	-10.4	-0.02 (0.07)	0.761
United Kingdom	59.0	61.5	4.1	-0.05 (0.08)	0.566
United States	98.2	100.9	2.7	-0.18 (0.12)	0.147

a No linear or quadratic trend
SE, standard error

Supplementary Table S2. Proportional beef and veal consumption as a % of total meat consumption in 2000 and 2019, presented for countries with decreasing, increasing and no temporal trend

Country	Beef and veal consumption, % of total meat consumption		Change in proportional beef and veal consumption		
	Year 2000	Year 2019	Change in % points (2000-2019)	Unstandardised β coefficient (SE)	P value
	%	%		% per year	
Decreasing linear trend					
Argentina	59.9	43.0	-16.9	-1.29 (0.11)	< 0.001
Australia	30.1	22.0	-8.1	-0.51 (0.05)	< 0.001
Brazil	40.3	31.9	-8.4	-0.71 (0.12)	< 0.001
Canada	30.6	25.1	-5.5	-0.25 (0.04)	< 0.001
Chile	29.9	24.7	-5.2	-0.28 (0.07)	0.001
Colombia	47.9	17.4	-30.5	-1.30 (0.07)	< 0.001
Egypt	45.8	39.0	-6.7	-0.29 (0.10)	0.007
India	43.8	14.9	-28.9	-1.88 (0.13)	< 0.001
Japan	24.5	18.1	-6.4	-0.21 (0.04)	< 0.001
Kazakhstan	52.6	40.1	-12.4	-0.57 (0.08)	< 0.001
Korea	24.8	19.1	-5.7	-0.17 (0.08)	0.045
Mexico	26.6	16.8	-9.8	-0.55 (0.03)	< 0.001
New Zealand	26.8	15.4	-11.4	-0.42 (0.11)	0.001
Norway	31.6	22.6	-8.9	-0.44 (0.04)	< 0.001
OECD	25.4	20.6	-4.7	-0.24 (0.01)	< 0.001
Paraguay	44.9	30.5	-14.3	-0.82 (0.31)	0.017
Peru	14.2	7.7	-6.5	-0.39 (0.02)	< 0.001
Philippines	14.8	9.7	-5.1	-0.26 (0.02)	< 0.001
Russia	34.8	16.1	-18.7	-0.99 (0.04)	< 0.001
South Africa	29.7	21.6	-8.1	-0.38 (0.04)	< 0.001
Switzerland	26.8	25.5	-1.3	-0.05 (0.02)	0.018
Thailand	10.5	6.5	-4.0	-0.12 (0.04)	0.008
Ukraine	41.7	11.5	-30.2	-1.37 (0.13)	< 0.001
United States	31.6	26.1	-5.5	-0.31 (0.02)	< 0.001
World	22.8	18.9	-3.9	-0.26 (0.02)	< 0.001
Increasing linear trend					
Ethiopia	70.2	80.7	10.5	0.81 (0.32)	0.020
Israel	21.0	25.7	4.7	0.28 (0.11)	0.021
Saudi Arabia	6.5	8.8	2.3	0.14 (0.04)	0.003
Turkey	21.5	28.9	7.4	0.65 (0.12)	< 0.001
Viet Nam	8.4	16.1	7.7	0.68 (0.07)	< 0.001
No trend detected[a]					
China (People's Republic of)	7.3	8.9	1.7	-0.01 (0.02)	0.745
Indonesia	20.8	19.0	-1.8	-0.06 (0.06)	0.303
Iran	16.3	17.2	0.9	-0.03 (0.04)	0.468

Malaysia	10.9	8.8	-2.1	-0.07 (0.04)	0.106
Nigeria	25.9	26.3	0.5	0.07 (0.09)	0.418
Pakistan	47.0	42.6	-4.4	-0.16 (0.11)	0.148
United Kingdom	18.0	18.6	0.6	0.03 (0.03)	0.409

a No linear or quadratic trend
SE, standard error

Supplementary Table S3. Beef consumption in 2000 and 2019 and % change, presented for countries with decreasing, increasing and no temporal trend

Country	Beef and veal consumption		Change in beef and veal consumption		
	Year 2000	Year 2019	% change (2000 - 2019)	Unstandardised β coefficient (SE)	P value
	Kg/capita	Kg/capita	%	Kg/capita/year	
Decreasing linear trend					
Argentina	45.3	38.0	-16.1	-0.29 (0.10)	0.008
Australia	26.5	19.7	-25.7	-0.39 (0.05)	< 0.001
Canada	23.4	17.6	-24.7	-0.32 (0.02)	< 0.001
Colombia	13.0	8.5	-34.3	-0.08 (0.04)	0.045
Ethiopia	3.1	2.5	-19.3	-0.04 (0.01)	< 0.001
India	1.3	0.5	-59.0	-0.05 (0.00)	< 0.001
Mexico	10.8	9.2	-15.0	-0.14 (0.02)	< 0.001
New Zealand	23.3	11.6	-50.1	-0.54 (0.07)	< 0.001
Nigeria	1.6	1.3	-17.8	-0.02 (0.01)	0.001
Norway	14.4	12.7	-12.2	-0.06 (0.02)	0.002
OECD	16.0	14.5	-9.4	-0.10 (0.01)	< 0.001
Paraguay	24.0	12.1	-49.7	-0.55 (0.19)	0.011
Switzerland	14.7	13.2	-10.3	-0.06 (0.02)	0.011
Thailand	2.2	1.2	-44.6	-0.03 (0.01)	0.002
Ukraine	11.9	4.6	-61.2	-0.27 (0.03)	< 0.001
United States	31.1	26.3	-15.3	-0.35 (0.04)	< 0.001
World	6.7	6.4	-4.6	-0.02 (0.01)	< 0.001
Increasing linear trend					
Chile	15.7	20.1	28.5	0.20 (0.05)	0.001
China (People's Republic of)	2.7	4.1	52.2	0.05 (0.01)	< 0.001
Indonesia	1.4	2.2	54.4	0.04 (0.004)	< 0.001
Iran	3.3	5.7	73.5	0.11 (0.01)	< 0.001
Israel	16.5	23.2	40.1	0.33 (0.11)	0.006
Kazakhstan	14.6	19.7	34.9	0.26 (0.04)	< 0.001
Korea	8.4	11.8	39.9	0.22 (0.04)	< 0.001
Malaysia	4.2	5.3	26.7	0.08 (0.02)	0.001
Pakistan	4.5	6.4	42.8	0.12 (0.01)	< 0.001
Peru	3.6	4.2	16.9	0.04 (0.01)	< 0.001
Saudi Arabia	2.9	3.8	34.3	0.08 (0.02)	0.001
South Africa	9.5	11.1	17.7	0.17 (0.03)	< 0.001
Turkey	4.0	9.5	139.0	0.39 (0.04)	< 0.001
Viet Nam	1.6	8.1	402.2	0.53 (0.04)	< 0.001
No trend detected[a]					
Brazil	25.2	25.2	-0.2	-0.13 (0.08)	0.108
Egypt	7.7	7.8	1.5	0.06 (0.03)	0.064
Japan	8.6	7.5	-12.5	-0.01 (0.02)	0.618
Philippines	3.4	3.2	-6.5	-0.01 (0.01)	0.058

Russia	10.3	10.1	-1.9	-0.08 (0.05)	0.140
United Kingdom	10.6	11.4	7.6	0.01 (0.02)	0.751

[a] No linear or quadratic trend

SE, standard error

Supplementary Table S4. Pork consumption in 2000 and 2019 and % change, presented for countries with decreasing, increasing and no temporal trend

Country	Pork consumption		Change in pork consumption		
	Year 2000	Year 2019	% change (2000 - 2019)	Unstandardised β coefficient (SE)	P value
	Kg/capita	Kg/capita	%	Kg/capita/year	
Decreasing linear trend					
Canada	22.6	16.3	-28.0	-0.33 (0.04)	< 0.001
Egypt	0.04	0.002	-94.3	-0.002 (0.0003)	< 0.001
Ethiopia	0.02	0.02	-11.8	-0.0001 (0.00004)	0.029
India	0.3	0.2	-51.0	-0.01 (0.0004)	< 0.001
Israel	1.9	1.3	-30.5	-0.04 (0.01)	< 0.001
Malaysia	5.8	5.3	-8.7	-0.05 (0.01)	0.001
Switzerland	26.4	22.5	-14.7	-0.24 (0.02)	< 0.001
Turkey	0.002	0.0	-100.0	0.00006 (0.00002)	0.001
United Kingdom	17.1	16.0	-6.2	-0.10 (0.02)	0.001
Increasing linear trend					
Argentina	6.0	11.4	89.9	0.35 (0.04)	< 0.001
Australia	15.7	20.3	29.4	0.30 (0.03)	< 0.001
Brazil	10.8	12.8	19.4	0.16 (0.03)	< 0.001
Chile	12.6	24.6	94.8	0.51 (0.06)	< 0.001
China (People's Republic of)	24.0	24.4	1.6	0.36 (0.08)	< 0.001
Colombia	2.1	9.0	321.6	0.35 (0.03)	< 0.001
Japan	13.3	16.2	21.2	0.10 (0.02)	< 0.001
Korea	16.0	31.2	95.7	0.75 (0.05)	< 0.001
Mexico	9.2	14.4	56.1	0.24 (0.02)	< 0.001
New Zealand	13.4	18.9	41.5	0.29 (0.03)	< 0.001
Nigeria	1.0	1.1	8.3	0.004 (0.002)	0.013
Norway	18.0	21.0	16.7	0.14 (0.02)	< 0.001
Peru	2.8	4.1	49.2	0.08 (0.01)	< 0.001
Philippines	12.5	15.2	21.8	0.12 (0.01)	< 0.001
Russia	9.5	20.6	115.6	0.57 (0.05)	< 0.001
Saudi Arabia	0.1	0.4	417.7	0.02 (0.004)	< 0.001
South Africa	2.1	3.7	76.3	0.08 (0.01)	< 0.001
Ukraine	11.9	12.9	8.5	0.23 (0.06)	0.001
Viet Nam	13.6	26.0	90.2	0.87 (0.09)	< 0.001
World	11.4	11.1	-2.6	0.05 (0.02)	0.006
No trend detected[a]					
Indonesia	1.5	1.0	-33.9	-0.02 (0.01)	0.117
Iran	0.0	0.0	-	-	-
Kazakhstan	4.5	4.7	5.2	-0.03 (0.03)	0.331
OECD	22.2	23.0	3.9	0.002 (0.02)	0.911
Pakistan	0.0	0.0	-	-	-

Paraguay	23.1	21.6	-6.8	0.12 (0.09)	0.192
Thailand	8.5	9.9	16.1	0.03 (0.03)	0.288
United States	23.4	24.0	2.5	-0.04 (0.04)	0.249

a No linear or quadratic trend
SE, standard error

Supplementary Table S5. Proportional pork consumption as a % of total meat consumption in 2000 and 2019, presented for countries with decreasing, increasing and no temporal trend

Country	Pork consumption % of total meat consumption		Change in proportional pork consumption		
	Year 2000	Year 2019	Change in % points (2000-2019)	Unstandardised β coefficient (SE)	P value
	%	%		% per year	
Decreasing linear trend					
Canada	29.5	23.2	-6.3	-0.28 (0.04)	< 0.001
China (People's Republic of)	65.1	53.3	-11.8	-0.28 (0.09)	0.005
Egypt	0.2	0.01	-0.2	-0.01 (0.002)	< 0.001
Indonesia	22.8	8.9	-13.9	-0.60 (0.15)	0.001
India	11.6	4.7	-6.9	-0.37 (0.02)	< 0.001
Israel	2.4	1.5	-1.0	-0.06 (0.01)	< 0.001
Kazakhstan	16.1	9.6	-6.5	-0.48 (0.04)	< 0.001
Malaysia	15.1	8.8	-6.3	-0.41 (0.03)	< 0.001
Norway	39.4	37.6	-1.8	-0.17 (0.04)	< 0.001
OECD	35.2	32.8	-2.3	-0.12 (0.01)	< 0.001
Peru	10.9	7.5	-3.4	-0.20 (0.02)	< 0.001
Philippines	54.9	47.0	-7.8	-0.52 (0.05)	< 0.001
Switzerland	48.3	43.7	-4.7	-0.32 (0.03)	< 0.001
Turkey	0.01	0.0	-0.01	-0.0003 (0.00009)	0.001
United Kingdom	29.0	26.1	-2.9	-0.14 (0.03)	< 0.001
Viet Nam	70.6	51.4	-19.2	-1.24 (0.15)	< 0.001
World	38.6	32.6	-6.0	-0.20 (0.03)	< 0.001
Increasing linear trend					
Argentina	7.9	12.9	5.0	0.29 (0.04)	< 0.001
Australia	17.7	22.6	4.9	0.27 (0.03)	< 0.001
Chile	24.1	30.2	6.1	0.20 (0.07)	0.010
Colombia	7.9	18.4	10.5	0.53 (0.05)	< 0.001
Ethiopia	0.4	0.5	0.1	0.01 (0.002)	0.002
Mexico	22.7	26.4	3.6	0.21 (0.03)	< 0.001
New Zealand	15.4	25.2	9.7	0.61 (0.04)	< 0.001
Nigeria	16.3	21.8	5.5	0.37 (0.03)	< 0.001
Paraguay	43.3	54.6	11.4	0.79 (0.25)	0.006
Russia	32.4	32.9	0.5	0.11 (0.04)	0.024
Saudi Arabia	0.2	0.9	0.8	0.04 (0.01)	0.001
No trend detected[a]					
Brazil	17.2	16.3	-0.9	-0.01 (0.04)	0.835
Iran	0.0	0.0			
Japan	38.0	38.9	0.9	-0.10 (0.05)	0.060

Korea	46.7	50.3	3.6	-0.01 (0.12)	0.963
Pakistan	0.0	0.0	0.0	-0.0001 (0.0001)	0.324
South Africa	6.5	7.1	0.6	-0.03 (0.02)	0.267
Thailand	40.1	52.0	11.9	0.24 (0.14)	0.102
Ukraine	41.7	32.2	-9.5	-0.26 (0.12)	0.050
United States	23.8	23.8	0.0	-0.0007 (0.02)	0.969

a No linear or quadratic trend,
SE, standard error

Supplementary Table S6. Poultry consumption in 2000 and 2019 and % change, presented for countries with decreasing, increasing and no temporal trend

Country	Poultry consumption		Change in poultry consumption		
	Year 2000	Year 2019	% change (2000 - 2019)	Unstandardised β coefficient (SE)	P value
	Kg/capita	Kg/capita	%	Kg/capita/year	
Decreasing linear trend					
Ethiopia	0.5	0.1	-77.5	-0.03 (0.005)	< 0.001
Nigeria	1.2	0.9	-25.6	-0.03 (0.01)	< 0.001
Paraguay	5.8	5.4	-7.6	-0.04 (0.02)	0.039
Increasing linear trend					
Argentina	22.9	37.9	65.6	1.30 (0.12)	< 0.001
Australia	29.7	43.5	46.5	0.80 (0.05)	< 0.001
Brazil	26.1	40.3	54.6	1.00 (0.11)	< 0.001
Canada	29.7	35.3	18.9	0.16 (0.04)	0.002
Chile	23.4	36.1	54.6	0.73 (0.05)	< 0.001
China (People's Republic of)	8.4	14.0	67.9	0.27 (0.02)	< 0.001
Colombia	11.7	31.3	167.4	1.02 (0.03)	< 0.001
Egypt	7.5	11.1	48.2	0.25 (0.03)	< 0.001
Indonesia	3.5	7.8	124.4	0.23 (0.01)	< 0.001
India	0.8	2.4	218.3	0.10 (0.002)	< 0.001
Iran	11.0	23.2	110.2	0.68 (0.04)	< 0.001
Japan	12.8	17.7	37.9	0.26 (0.02)	< 0.001
Kazakhstan	3.1	16.5	426.6	0.73 (0.06)	< 0.001
Korea	9.6	18.7	94.0	0.51 (0.04)	< 0.001
Malaysia	28.1	48.7	73.7	1.22 (0.04)	< 0.001
Mexico	19.8	30.5	53.9	0.50 (0.03)	< 0.001
New Zealand	24.6	41.1	67.4	0.59 (0.10)	< 0.001
Norway	8.5	17.7	108.0	0.54 (0.05)	< 0.001
OECD	22.9	31.3	36.8	0.38 (0.02)	< 0.001
Pakistan	2.1	6.6	211.9	0.24 (0.01)	< 0.001
Peru	17.7	45.3	156.8	1.62 (0.05)	< 0.001
Philippines	6.5	13.5	106.9	0.40 (0.02)	< 0.001
Russia	8.8	30.6	247.8	1.13 (0.04)	< 0.001
Saudi Arabia	34.5	34.9	1.2	0.24 (0.11)	0.044
South Africa	17.2	34.2	98.8	1.05 (0.05)	< 0.001
Switzerland	11.8	14.7	24.7	0.19 (0.03)	< 0.001
Turkey	9.3	19.1	105.6	0.58 (0.03)	< 0.001
Ukraine	4.4	22.3	405.4	0.92 (0.11)	< 0.001
United Kingdom	25.4	30.1	18.1	0.15 (0.06)	0.034
United States	43.2	50.1	15.9	0.22 (0.06)	0.002
Viet Nam	4.0	16.2	304.7	0.71 (0.07)	< 0.001
World	9.8	14.7	51.3	0.27 (0.01)	< 0.001

No trend detected[a]					
Israel	58.9	64.0	8.7	0.16 (0.09)	0.084
Thailand	10.4	7.8	-25.1	-0.03 (0.06)	0.638

a No linear or quadratic trend

SE, standard error

Supplementary Table S7. Proportional poultry consumption as a % of total meat consumption in 2000 and 2019, presented for countries with decreasing, increasing and no temporal trend

Country	Poultry consumption as a % of total meat consumption		Change in proportion of poultry consumption		
	Year 2000	Year 2019	Change in % points (2000-2019)	Unstandardised β coefficient (SE)	P value
	%	%		% per year	
Decreasing linear trend					
Ethiopia	11.28	3.62	-7.66	-0.47 (0.09)	< 0.001
Israel	74.91	71.10	-3.81	-0.24 (0.11)	0.041
Nigeria	18.69	17.22	-1.47	-0.17 (0.07)	0.030
Increasing linear trend					
Argentina	30.30	42.97	12.67	1.04 (0.11)	< 0.001
Australia	33.65	48.53	14.88	0.77 (0.04)	< 0.001
Brazil	41.62	51.11	9.48	0.73 (0.11)	< 0.001
Canada	38.82	50.33	11.52	0.51 (0.03)	< 0.001
China (People's Republic of)	22.67	30.68	8.01	0.22 (0.06)	0.002
Colombia	43.16	63.93	20.77	0.81 (0.08)	< 0.001
Egypt	44.57	55.47	10.90	0.58 (0.10)	< 0.001
Indonesia	51.53	68.31	16.78	0.83 (0.12)	< 0.001
India	25.46	67.46	42.01	2.58 (0.11)	< 0.001
Iran	54.86	70.05	15.19	0.90 (0.10)	< 0.001
Japan	36.57	42.58	6.01	0.34 (0.03)	< 0.001
Kazakhstan	11.27	33.61	22.33	1.13 (0.11)	< 0.001
Korea	28.20	30.09	1.90	0.16 (0.07)	0.027
Malaysia	72.64	80.67	8.03	0.45 (0.03)	< 0.001
Mexico	48.90	55.87	6.97	0.39 (0.05)	< 0.001
New Zealand	28.33	54.62	26.30	1.25 (0.07)	< 0.001
Norway	18.64	31.69	13.05	0.76 (0.07)	< 0.001
OECD	36.36	44.69	8.32	0.42 (0.01)	< 0.001
Pakistan	22.13	43.86	21.74	1.21 (0.04)	< 0.001
Peru	69.77	82.85	13.08	0.78 (0.04)	< 0.001
Philippines	28.62	41.67	13.06	0.78 (0.05)	< 0.001
Russia	29.88	48.96	19.08	0.90 (0.06)	< 0.001
South Africa	53.91	66.27	12.36	0.64 (0.05)	< 0.001
Switzerland	21.59	28.53	6.94	0.42 (0.03)	< 0.001
Ukraine	15.46	55.58	40.12	1.65 (0.22)	< 0.001
United Kingdom	43.12	48.92	5.80	0.28 (0.07)	0.001
United States	44.01	49.67	5.65	0.31 (0.02)	< 0.001
Viet Nam	20.72	32.11	11.39	0.56 (0.13)	< 0.001
World	33.05	43.36	10.32	0.49 (0.02)	< 0.001
No trend detected[a]					

Chile	44.62	44.43	-0.19	0.12 (0.06)	0.063
Paraguay	10.83	13.56	2.73	0.02 (0.08)	0.782
Saudi Arabia	78.41	80.12	1.71	0.06 (0.05)	0.288
Thailand	49.34	41.27	-8.07	-0.13 (0.16)	0.433
Turkey	50.30	58.11	7.81	0.14 (0.17)	0.398

a No linear or quadratic trend,

SE, standard error

Supplementary Table S8. Sheep meat consumption in 2000 and 2019 and % change, presented for countries with decreasing, increasing and no temporal trend

Country	Sheep meat consumption		Change in sheep meat consumption		
	Year 2000	Year 2019	% change (2000 - 2019)	Unstandardised β coefficient (SE)	P value
	Kg/capita	Kg/capita	%	Kg/capita/year	
Decreasing linear trend					
Argentina	1.4	1.0	-31.3	-0.02 (0.003)	< 0.001
Australia	16.4	6.2	-62.2	-0.44 (0.04)	< 0.001
Chile	0.7	0.5	-33.4	-0.01 (0.002)	< 0.001
Colombia	0.3	0.1	-51.0	-0.01 (0.006)	< 0.001
Egypt	1.6	1.1	-30.9	-0.03 (0.003)	< 0.001
Ethiopia	0.8	0.5	-41.2	-0.03 (0.01)	0.042
India	0.6	0.5	-19.2	-0.01 (0.0008)	< 0.001
Iran	5.8	4.2	-27.2	-0.11 (0.01)	< 0.001
Japan	0.3	0.2	-45.9	-0.01 (0.002)	< 0.001
Mexico	0.7	0.5	-23.7	-0.02 (0.003)	< 0.001
New Zealand	25.5	3.6	-85.9	-1.31 (0.13)	< 0.001
Nigeria	2.4	1.7	-28.5	-0.05 (0.01)	< 0.001
Norway	4.8	4.5	-4.7	-0.02 (0.01)	< 0.001
OECD	2.0	1.3	-34.6	-0.04 (0.003)	< 0.001
Pakistan	2.9	2.0	-31.2	-0.06 (0.01)	< 0.001
Peru	1.3	1.0	-18.4	-0.01 (0.001)	< 0.001
Saudi Arabia	6.6	4.4	-32.7	-0.08 (0.02)	< 0.001
Switzerland	1.8	1.2	-33.0	-0.03 (0.002)	< 0.001
United Kingdom	5.9	3.9	-32.8	-0.10 (0.01)	< 0.001
United States	0.5	0.4	-11.8	-0.004 (0.001)	0.027
Increasing linear trend					
China (People's Republic of)	1.8	3.2	78.1	0.07 (0.002)	< 0.001
Israel	1.3	1.5	20.4	0.02 (0.01)	0.001
Kazakhstan	5.6	8.2	47.0	0.17 (0.01)	< 0.001
Korea	0.1	0.3	213.2	0.01 (0.002)	< 0.001
Malaysia	0.5	1.0	91.6	0.04 (0.004)	< 0.001
Philippines	0.4	0.5	30.0	0.01 (0.002)	< 0.001
Russia	0.9	1.3	49.1	0.03 (0.003)	< 0.001
Thailand	0.01	0.04	200.0	0.002 (0.0001)	< 0.001
Viet Nam	0.1	0.2	207.5	0.005 (0.0007)	< 0.001
World	1.6	1.8	6.7	0.01 (0.001)	< 0.001
No trend detected[a]					
Brazil	0.6	0.5	-5.5	0.001 (0.0006)	0.073
Canada	0.8	1.0	17.4	0.0007 (0.003)	0.782
Indonesia	0.3	0.4	31.7	-0.003 (0.002)	0.244
Paraguay	0.5	0.5	-8.7	-0.001 (0.0008)	0.121
South Africa	3.2	2.6	-18.0	-0.01 (0.01)	0.333

Turkey	5.2	4.3	-17.8	-0.01 (0.02)	0.382
Ukraine	0.3	0.3	-8.6	-0.0006 (0.001)	0.737

a No linear or quadratic trend
SE, standard error

Supplementary Table S9. Proportional sheep meat consumption as a % of total meat consumption in 2000 and 2019, presented for countries with decreasing, increasing and no temporal trend

Country	Sheep meat consumption as a % of total meat consumption		Change in proportion of sheep meat consumption		
	Year 2000	Year 2019	Change in % points (2000-2019)	Unstandardised β coefficient (SE)	P value
	%	%		% per year	
Decreasing linear trend					
Argentina	1.87	1.10	-0.77	-0.05 (0.01)	< 0.001
Australia	18.55	6.90	-11.66	-0.53 (0.04)	< 0.001
Brazil	0.88	0.66	-0.22	-0.01 (0.001)	< 0.001
Chile	1.39	0.60	-0.80	-0.04 (0.003)	< 0.001
Colombia	1.07	0.29	-0.78	-0.04 (0.002)	< 0.001
Egypt	9.43	5.47	-3.96	-0.28 (0.02)	< 0.001
Indonesia	4.88	3.80	-1.08	-0.16 (0.03)	< 0.001
India	19.12	12.86	-6.26	-0.33 (0.03)	< 0.001
Iran	28.80	12.73	-16.07	-0.87 (0.08)	< 0.001
Japan	0.91	0.42	-0.50	-0.04 (0.005)	< 0.001
Mexico	1.76	1.00	-0.76	-0.06 (0.01)	< 0.001
New Zealand	29.40	4.78	-24.62	-1.44 (0.12)	< 0.001
Nigeria	39.20	34.68	-4.52	-0.27 (0.05)	< 0.001
Norway	10.42	8.12	-2.30	-0.15 (0.01)	< 0.001
OECD	3.10	1.82	-1.28	-0.06 (0.004)	< 0.001
Pakistan	30.91	13.52	-17.38	-1.05 (0.10)	< 0.001
Peru	5.05	1.90	-3.14	-0.19 (0.01)	< 0.001
Russia	2.90	2.04	-0.86	-0.02 (0.01)	0.004
Saudi Arabia	14.93	10.15	-4.78	-0.24 (0.04)	< 0.001
South Africa	9.89	5.02	-4.87	-0.23 (0.02)	< 0.001
Switzerland	3.23	2.29	-0.94	-0.04 (0.01)	< 0.001
Turkey	28.17	13.00	-15.17	-0.79 (0.09)	< 0.001
Ukraine	1.06	0.69	-0.37	-0.03 (0.003)	< 0.001
United Kingdom	9.92	6.40	-3.51	-0.16 (0.02)	< 0.001
United States	0.51	0.44	-0.07	-0.003 (0.001)	0.032
World	5.56	5.15	-0.41	-0.03 (0.00)	< 0.001
Increasing linear trend					
Canada	1.09	1.39	0.30	0.01 (0.003)	0.007
China (People's Republic of)	4.93	7.08	2.15	0.07 (0.01)	< 0.001
Israel	1.60	1.69	0.08	0.02 (0.01)	0.021
Korea	0.31	0.53	0.22	0.01 (0.003)	0.003
Malaysia	1.41	1.73	0.32	0.03 (0.01)	< 0.001
Thailand	0.07	0.22	0.16	0.01 (0.0005)	< 0.001

No trend detected[a]					
Ethiopia	18.13	15.19	-2.94	-0.34 (0.25)	0.183
Kazakhstan	20.05	16.68	-3.37	-0.08 (0.05)	0.093
Paraguay	1.01	1.25	0.24	0.01 (0.01)	0.132
Philippines	1.70	1.55	-0.14	0.001 (0.01)	0.858
Viet Nam	0.27	0.32	0.05	-0.002 (0.002)	0.305

a No linear or quadratic trend,
SE, standard error

Supplementary Table S10. Hierarchical cluster analysis agglomeration schedule of countries based on change in gdp per capita and change in total meat consumption between the years 2000 and 2019

	Cluster Combined			Stage Cluster First Appears		
Stage	Cluster 1	Cluster 2	Coefficients	Cluster 1	Cluster 2	Next Stage
1	157	166	0.002	0	0	8
2	164	169	0.005	0	0	10
3	156	158	0.008	0	0	7
4	152	172	0.012	0	0	14
5	151	165	0.015	0	0	11
6	155	175	0.022	0	0	20
7	156	167	0.032	3	0	9
8	157	159	0.043	1	0	13
9	156	161	0.064	7	0	27
10	160	164	0.088	0	2	17
11	151	162	0.115	5	0	20
12	153	170	0.142	0	0	19
13	157	171	0.183	8	0	21
14	152	154	0.231	4	0	22
15	178	185	0.285	0	0	24
16	177	182	0.343	0	0	25
17	160	173	0.405	10	0	22
18	180	184	0.489	0	0	26
19	153	168	0.576	12	0	23
20	151	155	0.667	11	6	23
21	157	176	0.831	13	0	27
22	152	160	1.068	14	17	31
23	151	153	1.307	20	19	28
24	178	183	1.609	15	0	30
25	177	179	1.932	16	0	32
26	180	181	2.307	18	0	30
27	156	157	2.709	9	21	31
28	151	163	3.122	23	0	29
29	151	174	3.716	28	0	33
30	178	180	4.966	24	26	32

31	152	156	7.056	22	27	33
32	177	178	10.280	25	30	34
33	151	152	18.172	29	31	34
34	151	177	33.450	33	32	0

Supplementary Table S11. Assignment of countries into clusters based on change in gdp per capita and change in total meat consumption between the years 2000 and 2019 using hierarchical cluster analysis

	Total meat	Beef and veal	Pork	Poultry	Sheep meat
Location	Cluster number				
AUS	2	2	2	2	2
CAN	2	2	2	2	2
CHE	2	2	2	2	2
GBR	2	1	1	1	1
ISR	2	2	2	2	2
NOR	2	2	2	2	2
NZL	2	2	2	2	2
SAU	2	2	1	1	2
USA	2	2	2	2	2
ARG	1	1	1	1	1
BRA	1	1	1	1	1
CHL	1	1	1	1	1
CHN	1	1	1	1	1
COL	1	1	1	1	1
EGY	1	1	1	1	1
ETH	1	1	1	1	1
IDN	1	1	1	1	1
IND	1	1	1	1	1
IRN[a]	1	1	-	1	1
JPN	1	1	1	1	1
KAZ	1	1	1	1	1
KOR	1	2	1	2	2
MEX	1	1	1	1	1
MYS	1	1	1	1	1
NGA	1	1	1	1	1
PAK	1	1	1	1	1
PER	1	1	1	1	1
PHL	1	1	1	1	1
PRY	1	1	1	1	1
RUS	1	1	1	1	1
THA	1	1	1	1	1
TUR	1	1	1	1	1
UKR	1	1	1	1	1
VNM	1	1	1	1	1
ZAF	1	1	1	1	1

[a] IRN (Iran) was placed in neither cluster for pork because consumption was zero

NOTES: Argentina (ARG), Australia (AUS), Brazil (BRA), Canada (CAN), Switzerland (CHE), Chile (CHL), People's Republic Of China (CHN), Colombia (COL), Arab Republic of Egypt (EGY), Ethiopia (ETH), United Kingdom (GBR), India (IND), Indonesia (IDN), Islamic Republic of Iran (IRN), Israel (ISR), Japan (JPN), Kazakhstan (KAZ), Republic of Korea (KOR), Mexico (MEX), Malaysia (MYS), Nigeria (NGA), Norway (NOR), New Zealand (NZL), Pakistan (PAK), Peru (PER), Philippines (PHL), Paraguay (PRY), Russian Federation (RUS), Saudi Arabia (SAU), Thailand (THA), Turkey (TUR), Ukraine (UKR), United States (USA), Viet Nam (VNM) and South Africa (ZAF).

Supplementary Figure S1. Hierarchical cluster analysis dendrogram (using Ward linkage) of countries based on change in GDP per capita and change in total meat consumption between the years 2000 and 2019

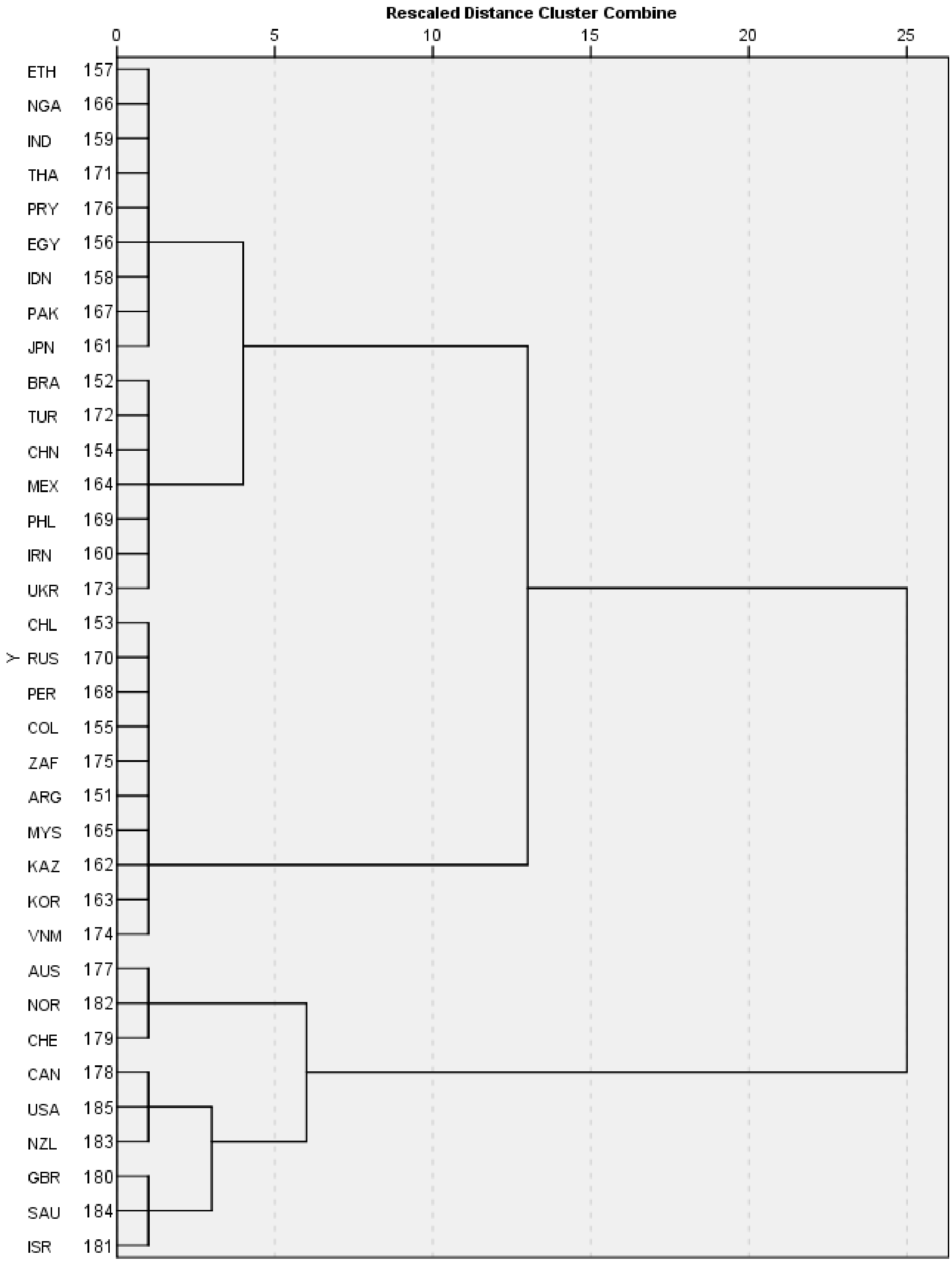

NOTES: Argentina (ARG), Australia (AUS), Brazil (BRA), Canada (CAN), Switzerland (CHE), Chile (CHL), People's Republic of China (CHN), Colombia (COL), Arab Republic of Egypt (EGY), Ethiopia (ETH), United Kingdom (GBR), India (IND), Indonesia (IDN), Islamic Republic of Iran (IRN), Israel (ISR), Japan (JPN), Kazakhstan (KAZ), Republic of Korea (KOR), Mexico (MEX), Malaysia (MYS), Nigeria (NGA), Norway (NOR), New Zealand (NZL), Pakistan (PAK), Peru (PER), Philippines (PHL), Paraguay (PRY), Russian Federation (RUS), Saudi Arabia (SAU), Thailand (THA), Turkey (TUR), Ukraine (UKR), United States (USA), Viet Nam (VNM), and South Africa (ZAF).
